# Fatty acid metabolism in breast cancer subtypes

**DOI:** 10.18632/oncotarget.15494

**Published:** 2017-02-18

**Authors:** Marie E. Monaco

**Affiliations:** ^1^ Department of Neuroscience & Physiology, New York University School of Medicine, New York, NY, USA; ^2^ Veterans Affairs New York Harbor Healthcare System, New York, NY, USA

**Keywords:** fatty acid metabolism, molecular subtype, breast cancer

## Abstract

Dysregulation of fatty acid metabolism is recognized as a component of malignant transformation in many different cancers, including breast; yet the potential for targeting this pathway for prevention and/or treatment of cancer remains unrealized. Evidence indicates that proteins involved in both synthesis and oxidation of fatty acids play a pivotal role in the proliferation, migration and invasion of breast cancer cells. The following essay summarizes data implicating specific fatty acid metabolic enzymes in the genesis and progression of breast cancer, and further categorizes the relevance of specific metabolic pathways to individual intrinsic molecular subtypes of breast cancer. Based on mRNA expression data, the less aggressive luminal subtypes appear to rely on a balance between *de novo* fatty acid synthesis and oxidation as sources for both biomass and energy requirements, while basal-like, receptor negative subtypes overexpress genes involved in the utilization of exogenous fatty acids. With these differences in mind, treatments may need to be tailored to individual subtypes.

## INTRODUCTION

Fatty acid metabolism (FAM) comprises a variety of pathways including fatty acid transport, *de novo* synthesis, activation, incorporation into neutral and polar glycerides, storage as triglyceride and cholesterol esters in lipid droplets, mobilization from phospholipids and triglycerides, oxidation and, in the case of arachidonic acid, conversion to eicosanoids such as prostaglandins and leukotrienes. The malignant phenotype is characterized by alterations in these pathways, and the potential for targeting these pathways in the prevention and treatment of breast cancer is well recognized, although not yet realized.

To date, most investigations into the role of FAM in breast cancer have failed to take into account the heterogeneous nature of the disease. There has been little effort to define alterations in FAM pathways with respect to intrinsic molecular subtype. Generalizations concerning fatty acid metabolism in breast cancer, therefore, are likely derived from, and characteristic of, the most prevalent forms of the disease, i.e., estrogen receptor alpha (ER) positive subtypes.

In the following essay we review data relevant to the role of altered FAM in sustaining the proliferation and progression of breast cancer, with emphasis on pathways that appear specific for individual molecular subtypes. Published data is accompanied by a meta-analysis of public gene expression data that suggest that future approaches to exploit FAM pathways in the prevention and treatment of breast cancer should be tailored to individual molecular subtypes.

## PATTERNS OF EXPRESSION FOR FAM GENES DIFFER BY MOLECULAR SUBTYPE

Within the last 15 years, a number of studies have utilized mRNA expression patterns to subtype breast cancers according to molecular signatures that might predict clinical outcomes [1]. Although these subtypes are generally comparable to receptor status as determined by immunostaining (ER, progesterone receptor (PR) and human epidermal growth factor receptor 2 (HER2)), there are, nevertheless, refinements that have proved useful. In the case of the PAM50 molecular signature [2], which utilizes a 50-gene mRNA expression panel, tumors are classified as luminal A (ER+, PR+), luminal B (ER+, PR+ or PR-), HER2-enriched (HER2+), basal-like (ER-, PR-, androgen receptor negative (AR-)) and normal-like [3]. Whether the normal-like category is a distinct breast cancer subtype or reflects contamination with normal cells is not yet clear [4]. The luminal A, receptor positive, subtype has the best prognosis. Triple negative breast cancers (TNBC), designated as such by immunostaining, fall predominantly (79%) into the basal-like category, with 8% HER2-enriched, 7% luminal A and luminal B and 7% normal-like [4]. It has already been determined that AR+ TNBC are markedly different and should be considered as a separate category [4–6]. AR+ TNBC can fall into either the luminal or HER2-enriched category. It has been suggested that AR- TNBC be referred to as quadruple negative breast cancer (QNBC) [7, 8].

**Figure 1:**
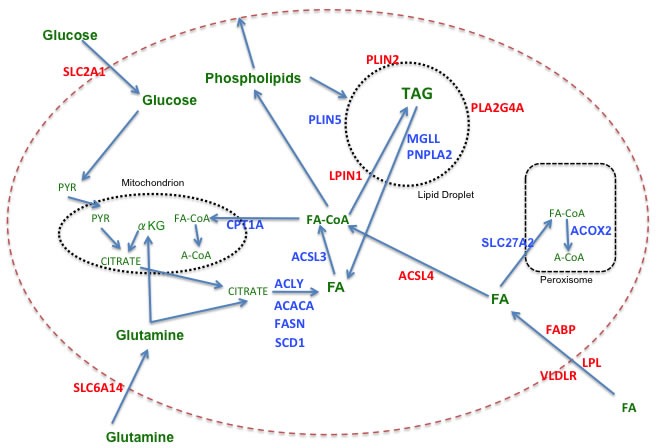
Fatty acid metabolism in breast cancer Color code: Red = genes more highly expressed in TNBC versus RPBC; Blue = genes more highly expressed in RPBC versus TNBC; Green = metabolic substrates. Abbreviations: ACACA: acetyl-CoA carboxylase alpha; ACLY: ATP citrate lyase; ACOX2: acyl-CoA oxidase 2, branched chain; ACSL: long chain fatty acyl-CoA synthetase; CPT1A: carnitine palmitoyltransferase 1A; FABP: fatty acid binding protein; FASN: fatty acid synthase; LPIN1: lipin 1 (also known as phosphatidic acid phosphohydralase (PAP); LPL: lipoprotein lipase; MGLL: monoglyceride lipase; PLA2G4A: phospholipase A2, group IVA (also known as cytosolic phospholipase A2 (cPLA2);PLIN: perilipin; PNPLA2: palatin-like phospholipase domain containing 2 (also known as ATGL, adipocyte triglycderide lipase); SCD1: stearoyl-CoA desaturase 1; SLC2A1: Solute carrier family 2 member 1 (also know as GLUT1, glucose transporter 1); SLC6A14: solute carrier family 6 (amino acid transporter) member 14; SLC27A2: solute carrier family 27 member 2 (also known as ACSVL1, very long chain fatty acyl-CoA synthetase 1), FA: fatty acid; PYR: pyruvate; αKG: alpha ketoglutarate; A-CoA: acetylCoA; FA-CoA: fatty acyl-CoA.

Figure 1 depicts pathways of FAM, including the relationship of FAM to glucose and glutamine metabolism, highlighting aspects found to be relevant to sustaining growth and survival of breast cancer cells. In a survey of gene expression data on the Oncomine site [9], relative mRNA expression levels for entities that are preferentially expressed in TNBC *versus* receptor-positive breast cancers (RPBC) are depicted in red, while those preferentially expressed in RPBC are in blue. Table 1a is an overview of the results from 21 different studies comparing expression in TNBC with that in RPBC. Table 1b details the differences as demonstrated in results from 6 of those studies: 5 involving tissue samples [10–14] and one utilizing breast cancer cell lines [15]. While these data are not necessarily reflective of protein expression or activity levels, they nevertheless suggest a clear-cut trend indicating that TNBC, in addition to relying more heavily on uptake of glucose (SLC2A1) and glutamine (SLC6A14), also appear to be more dependent on uptake and storage of exogenous fatty acids than RPBC, which instead up regulates *de novo* fatty acid synthesis, mobilization and oxidation to a greater degree than TNBC. This conclusion has been confirmed in a recent report of a lipid metabolism gene signature comparing TNBC to RNBC [16]. A comparison of the cell line data from Table 1b with the tissue data indicates that the preferential expression of many of the FAM genes observed *in vivo* is recapitulated in *in vitro* models. Note that these data do not address the question of expression relative to normal breast. Normal luminal epithelial cells differ from luminal breast cancers in that only a minority of the normal cells are ER+ (15-30%) [17], and cell lines derived from normal breast are most closely aligned with the basal-like, receptor-negative subtype [18].

**Table 1A:** Overview of fatty acid metabolism gene mRNA expression in breast cancer

		TNBC relative to RPBC				
		CHANGE (P<0.05)		CHANGE (NS)		NO. of STUDIES
		↑	↓	↑	↓	
	GENE					
**FUNCTION**						
**Synthesis**	ACLY	0	9	3	9	21
	ACACA	0	13	2	6	21
	FASN	0	14	3	4	21
	SCD1	0	12	0	9	21
	SREBP1	0	19	0	2	21
**Transport**	VLDLR	15	0	4	1	20
	LPL	10	0	8	3	21
	FABP5	18	0	2	1	21
	FABP7	15	0	5	1	21
**Activation**	ACSL3	1	14	1	4	20
	ACSL4	15	0	5	1	21
	SLC27A2	0	17	1	2	21
**Storage**	PLIN2	18	0	2	1	21
	PLIN5	0	8	0	5	13
	LPIN1	19	0	1	1	21
**Mobilization**	MMGL	0	14	2	5	21
	PNPLA2	1	11	3	6	21
	PLA2G4A	16	0	4	1	21
**Oxidation**	CPT1A	1	16	1	3	21
	ACOX2	0	20	1	0	21
**Uptake of**						
**Glucose**	SLC2A1	11	1	8	1	21
**Glutamine**	SLC6A14	15	0	1	2	18

Data taken from Oncomine [9].

**Table 1B:** Fatty acid metabolism in TNBC compared with RPBC

		STUDY											
		Chin (*n*=19, 87)		Curtis (*n* =211, 1340)		Hatzis (*n* =178, 320)		Richardson2 (*n* =18, 19)		TCGA (*n* =46, 250)		Neve (*n* =21, 25)	
		FC	pV	FC	pV	FC	pV	FC	pV	FC	pV	FC	pV
	**GENE**												
**FUNCTION**													
**Synthesis**	ACLY	−1.25	0.013	−1.22	2.56e-14	−1.08	0.008	−1.53	0.007	−1.13	0.019	−1.16	0.092
	ACACA	−1.22	1.30e-05	−1.21	1.81e-09	−1.08	1.12e-04	−1.49	0.038	−1.25	0.003	−1.14	0.014
	FASN	−1.86	0.003	−1.60	7.83e-16	−1.72	2.33e-15	−6.37	3.12e-08	−2.22	1.20e-14	−2.11	1.36e-04
	SCD1	−1.65	0.006	−1.27	1.15e-06	−1.52	6.25e-08	−2.17	0.004	−2.23	1.07e-07	−1.82	0.002
	SREBP1	−1.81	3.91e-05	−1.85	5.46e-38	−1.66	1.37e-26	−2.76	2.82e-04	−2.18	1.14e-14	−1.49	0.001
**Transport**	VLDLR	1.54	0.007	1.67	1.14e-29	1.44	2.12e-17	2.33	0.022	2.26	8.47e-10	1.35	0.043
	LPL	1.45	0.049	1.28	9.54e-07	1.31	1.85e-04	3.74	0.001	1.47	0.028	1.18	0.079
	FABP5	2.29	3.05e-06	2.47	5.66e-44	2.60	1.15e-23	4.38	1.48e-05	3.58	3.59e-21	5.41	0.007
	FABP7	8.27	2.86e-04	2.80	0.013	2.37	7.49e-14	78.03	1.41e-07	23.50	6.59e-16	−1.39	0.08
**Activation**	ACSL3	−1.69	6.22e-04	−1.16	2.21e-07	−1.38	1.25e-07	−1.43	0.044	−1.39	6.75e-04	−1.69	0.007
	ACSL4	1.40	8.46e-04	1.43	1.13e-28	1.13	5.93e-10	1.84	0.002	1.64	8.40e-09	1.92	1.90e-05
	SLC27A2	−2.10	5.72e-09	−2.04	8.15e-80	−1.54	4.03e-18	−1.64	0.008	.4.00	9.70e-27	−1.32	0.045
**Storage**	PLIN2	1.68	2.22e-05	1.94	6.02e-36	1.88	6.81e-21	1.70	0.016	2.17	8.13e-14	1.62	0.023
	PLIN5	NA	NA	−1.10	4.15e-27	NA	NA	−1.24	0.007	−3.20	6.89e-10	NA	NA
	LPIN1	2.43	4.76e-08	2.19	8.21e-55	1.85	6.62e-27	3.16	2.64e-06	2.98	1.40e-15	1.34	0.014
**Mobilization**	MGLL	−1.81	1.64e-04	−1.25	4.11e-10	−1.24	1.26e-06	−2.43	0.003	−1.64	2.75e-05	−1.39	0.051
	PNPLA2	−1.06	0.219	−1.32	8.56e-25	−1.05	0.002	−1.18	0.046	−1.30	9.83e-06	−1.10	0.011
	PLA2G4A	3.13	9.26e-05	1.06	6.31e-09	1.33	4.11e-09	6.47	1.69e-04	3.91	3.07e-12	1.38	0.055
**Oxidation**	CPT1A	−1.65	4.08e-05	−1.07	4.78e-12	−1.20	5.80e-09	−2.21	0.001	−1.72	4.05e-10	−2.46	6.51e-07
	ACOX2	−4.11	1.36e-18	−3.97	8.20e-74	−1.62	1.74e-23	−7.37	1.89e-04	−5.87	5.82e-23	−1.61	0.009
**Uptake of**													
**Glucose**	SLC2A1	1.56	0.004	1.79	2.80e-26	1.14	5.40e-07	1.86	0.003	1.74	4.73e-07	1.07	0.908
**Glutamine**	SLC6A14	2.44	0.003	1.29	1.12e-16	1.84	2.00e-14	9.78	2.78e-04	15.74	2.69e-17	1.01	0.476

Data taken from 6 separate studies as reported on Oncomine [9]. The numbers in parentheses indicate sample size (n=TNBC, RPBC)

Abbreviations: FC = fold change (log 2 median-centered intensity)

pV = p value

NA = not available

As detailed above, the TNBC designation, although similar to, is not synonymous with classification of breast tumors according to gene signatures, such as PAM50 [19]. To determine whether the pattern suggested by the data shown in Table 1 persisted when samples were grouped by PAM50 subtype rather than receptor status, the expression of the same genes was evaluated as a function of subtype in 3 separate studies detailed in the Oncomine database [11, 12, 20]. Table 2 details the results, which reflect the relative degree of expression of a particular mRNA in a given subtype. The results generally mirror those obtained when expression is classified as a function of receptor status (Table 1). Note that each study utilizes a different microarray platform, which might explain minor differences between studies. Thus, whether utilizing receptor status or PAM50 classification, clear differences are observed with respect to relative mRNA expression levels for FAM proteins, with *de novo* fatty acid synthesis and oxidation predominating in RPBC, while uptake and storage of exogenous fatty acids predominate in QNBC.

**Table 2:** Expression of fatty acid metabolism genes as a function of intrinsic molecular subtype (PAM50)

		STUDY														
		Curtis(*n*=1986) Illumina					Esserman (*n*=129) Agilent					Hatzis (*n*=508) Affymetrix				
		Rank					Rank					Rank				
**FUNCTION**	GENE	1	2	3	4	5	1	2	3	4	5	1	2	3	4	5
																
**Synthesis**	ACLY	L	H	A	B	N	H	A	L	B	N	H	L	A	B	N
	ACACA	N	B	A	L	H	L	A	H	B	N	H	L	A	N	B
	FASN	H	A	L	N	B	L	H	A	B	N	L	A	H	N	B
	SCD1	H	L	A	N	B	H	N	B	A	L	H	L	N	A	B
	SREBP1	A	L	H	N	B	L	N	A	H	B	A	L	H	N	B
**Transport**	VLDLR	B	H	A	L	N	B	N	H	A	L	B	H	N	A	L
	LPL	N	B	A	L	H	N	B	H	A	L	N	B	A	H	L
	FABP5	B	N	L	H	A	B	H	N	L	A	B	N	H	L	A
	FABP7	B	N	H	A	L	N	B	H	A	L	B	N	H	A	L
**Activation**	ACSL3	H	N	L	A	B	L	A	N	B	H	L	H	A	N	B
	ACSL4	B	N	H	A	L	B	H	L	N	A	B	N	H	L	A
	SLC27A2	L	A	N	H	B	A	L	N	H	B	A	L	H	N	B
**Storage**	PLIN2	B	N	H	L	A	B	H	L	N	A	B	H	N	L	A
	PLIN5	A	L	N	H	B	L	A	N	B	H	na	na	na	na	na
	LPIN1	B	N	H	A	L	N	B	H	L	A	B	N	H	L	A
**Mobilization**	MGLL	N	H	A	L	B	N	A	H	L	B	N	H	A	L	B
	PNPLA2	A	N	L	H	B	A	H	L	B	N	N	A	B	L	H
	PLA2G4A	B	N	A	L	H	B	H	N	A	L	B	N	H	A	L
**Oxidation**	CPT1A	H	B	L	N	A	H	L	A	N	B	L	H	A	N	B
	ACOX2	A	L	H	N	B	A	N	L	H	B	L	A	N	A	B
**Uptake of**																
**glucose**	SLC2A1	B	H	L	A	N	B	L	H	A	N	B	H	N	A	L
**glutamine**	SLC6A14	B	N	H	A	L	B	H	L	N	A	B	N	H	A	L

Data taken from Oncomine [9]. Subtypes with highest and lowest expression indicated as rank 1 to 5, respectively. Abbreviations: n = sample number; A=luminal A; L=luminal B;H=HER2-enriched; B=basal-like; N=normal-like. The designation under the sample number refers to the microarray platform

Lastly, to further assess the importance of individual FAM proteins in mediating an aggressive breast cancer phenotype, we utilized public mRNA expression data [21] to determine the effect of silencing ER on expression of mRNA for FAM proteins in MCF7 cells. Results are shown in Table 3. Expression levels of mRNA for a number of proteins more highly expressed in TNBC were increased following silencing of ER, including VLDLR, FABP5, ACSL4, PLIN2, and PLA2G4A, while expression of some of those more highly expressed in RPBC was significantly decreased, including ACACA, FASN, SCD1, ACSL3, SLC27A2, PLIN5, MGLL and CPT1A. Of the 17 proteins that are expressed in MCF7 cells, 13 responded to ablation of ER activity with the expected change predicted by expression patterns summarized in Table 1. Considering that there are most likely major differences between the environment of tumor cells *in vivo* and *in vitro*, as well as the probability that there are also significant differences between naturally arising TNBC and forced ablation of ER only, the degree of symmetry is strong evidence of the validity of the analysis. These data further support the idea that expression patterns of FAM proteins are aligned with intrinsic molecular subtypes in breast cancer.

**Table 3:** Effect of ERα silencing on expression of fatty acid metabolic genes

		Control	Silenced	Fold Change	*p* Value
**Function**	**Gene**				
**Synthesis**	ACLY	1201±117	1896±146	1.57	0.003
	ACACA	387±17	95±2	−4.07	9.08e-06
	FASN	2863±353	942±327	−3.03	0.002
	SCD1	7181±86	3606±281	−1.99	3.02e-05
	SREBP1	941±65	383±34	−2.45	1.90e-04
**Transport**	VLDLR	248±3	383±45	1.54	0.007
	LPL	NE	NE		
	FABP5	1566±54	4101±269	2.61	8.94e-05
	FABP7	NE	NE		
**Activation**	ACSL3	2043±41	1684±96	−1.21	0.004
	ACSL4	150±5	1867±43	12.44	2.30e-06
	SLC27A2	913±60	18±1	−50.72	1.39e-05
**Storage**	PLIN2	95±9	575±86	6.05	6.70e-04
	PLIN5	136±7	46±5	−2.95	8.38e-05
	LPIN1	86±6	69±22	−1.24	0.266
**Mobilization**	MGLL	948±17	344±15	−2.76	1.39e-06
	PNPLA2	170÷18	186±34	1.09	0.534
	PLA2G4A	10±1	127±7	12.70	1.14e-05
**Oxidation**	CPT1A	141±7	23±2	−6.13	1.06e-05
	ACOX2	96±7	759±61	7.90	5.03e-o5
**Uptake of**					
**Glucose**	SLC2A1	629±37	3090±89	4.91	1.61e-06
**Glutamine**	SLC6A14	301±10	23±2	−13.08	1.33e-06

Data shown are mRNA expression values in arbitrary units.

Abbreviations: NE = not expressed

Fold Change = Silenced/Control for positive changes;

= −1 x Control/Silenced for negative changes

Published data relative to the role of individual FAM proteins in breast cancer is summarized below.

## DE NOVO FATTY ACID SYNTHESIS

In most human tissues, the *de novo* pathway of fatty acid synthesis is of minor importance, the exceptions being liver and mammary gland, and, to a lesser extent, adipose tissue [22]. The cellular requirement for fatty acids is generally met by utilization of dietary fatty acids. The differential importance of this pathway with respect to normal *versus* cancer tissue makes it an attractive target for therapy.

### ATP citrate lyase (ACLY)

As noted in Figure 1, the acetyl CoA required for fatty acid synthesis is derived from citrate *via* catalysis by the enzyme ACLY. Increased ACLY expression and activity have been noted in a number of cancers, including breast, leading to the suggestion that it is a key player in cancer metabolism [23]. This enzyme appears to be elevated in breast malignancies [24], and its inhibition by RNAi or the chemical inhibitor SB-204990 has a negative effect on proliferation and growth both *in vitro* and *in vivo* [25]. Steeg *et. al*. have suggested that phosphorylation of ACLY by the Nm23-H1 metastasis suppressor may play a role in limiting migration and invasion of tumor cells [26]. It has recently been reported that ACLY is required for cyclin E-mediated transformation, migration and invasion of breast cancer cells *in vitro*, as well as for tumor growth *in vivo* [27]*.* ACLY mRNA is most highly expressed in the HER2-enriched subtype, and therefore underexpressed in TNBC relative to RPBC.

### Acetyl CoA carboxylase alpha (ACACA)

The rate-limiting step in the synthesis of fatty acids is the ATP-dependent conversion of acetyl CoA to malonyl CoA by the enzyme, acetyl CoA carboxylase (ACAC). Two isoforms have been identified, ACACA (also called ACC1) and ACACB (also called ACC2). A number of studies have implicated ACACA activity in the malignant phenotype of breast cancer. An early immunohistochemical study of human breast tissue samples noted that expression of both ACAC isoforms was elevated upon development of either *in situ* duct or lobular carcinoma [28]. More recently, it has been demonstrated that silencing of the ACACA gene in human breast cancer cells in long-term culture as a result of RNA interference results in decreased palmitic acid synthesis and the induction of apoptosis [29]. Data also suggest that HER2 overexpression plays a role in increasing ACACA activity [30]. Breast cancer cell lines that overexpress HER2 (SK-BR-3 and BT-474) express higher levels of ACACA than those in which expression of HER2 is relatively low (MCF-7 and MDA-MB-231). Exogenous expression of HER2 in MDA-MB-231 cells induced expression of ACACA that appeared to be mediated at the translational level by mTOR *via* the PI3K/Akt pathway. And when MCF7 cells were engineered to overexpress HER2 they became 5 times more sensitive to ACACA inhibition-induced reductions in mammosphere-forming efficiency [31]. The mRNA data for ACACA indicate that expression is decreased in TNBC relative to RNBC (Table 1A and 1B) in both tissue samples and cell lines, and that silencing of ER in MCF7 cells decreases expression by 4-fold (Table 3).

### Fatty acid synthase (FASN)

The most extensively studied of the lipogenic enzymes in the context of carcinogenesis is FASN (for reviews, see [32–36]). Overexpression of this enzyme has been reported for a variety of cancers, including prostate, liver, ovary, colon, endometrium and breast, and has been associated with malignant transformation and a worse prognosis. Using the h-ras-induced transformation of the human mammary epithelial cell line, MCF10A, as a model, Yang *et. al.* demonstrated that upregulation of FASN activity was driven by increases in EGF signaling *via* a pathway including MAPK, PI3K and sterol regulatory element binding protein 1 (SREBP1). Inhibitors of the kinases lowered SREBP1 levels and decreased FASN transcription. Deletion of the SREBP binding site from the FAS promoter also decreased FAS transcription in the transformed cells [37]. HER2 is another member of the erbB2 family of receptor tyrosine kinases that has been demonstrated to play a role in regulation of FASN activity [38]. A transcriptome analysis comparing HER2-positive with HER2-negative breast cancer cell lines demonstrated that expression of FASN was increased in cells that were HER2-positive. Inhibition of HER2 activity with either Herceptin or a kinase inhibitor resulted in a reduction of FASN expression. HER2 was shown to stimulate the FAS promoter *via* a mechanism involving PI3K that ultimately resulted in increase incorporation of radiolabeled acetate into fatty acids [38]. More recently HER2 has also been shown to directly phosphorylate FASN, which results in activation of FASN activity [39]. Inhibition of FASN phosphorylation decreased invasion by SK-BR-3 and BT-474 breast cancer cells. FASN has also been suggested to play the role of an oncogene in cell transformation. Forced overexpression of FASN in HBL100 and MCF-10A cells caused these near normal breast cells to exhibit transformed properties and to increase expression levels of EGFR and HER2 [40]. When FASN mRNA expression is correlated with molecular subtype, there is a clear association between FASN and RPBC, such as HER2-enriched, luminal A and luminal B, as shown in the 3 studies analyzed in Table 2. Immunochemical analysis of tumor samples confirms that FASN protein expression is highest in HER2 enriched tumors, and lowest in TNBC [41]. Like FASN, SREBP1 is preferentially expressed in RPBC (Tables 1 and 2).

### Stearoyl-CoA desaturase 1 (SCD1)

The product of FASN activity is the long chain saturated fatty acids (SFA); however, in many instances the cell converts these SFAs to monounsaturated fatty acids before subsequent utilization [42]. The enzyme responsible for catalyzing the introduction of a double bond in the *cis*-delta-9 position is a fatty acyl-CoA delta 9 desaturase, also known as stearoyl-CoA desaturase (SCD). Two isoforms of SCD have been reported in human cells, SCD1 and SCD5 [43, 44]. It has been suggested that SCD1 may play a key role in the generation of the malignant phenotype, as well as in the subsequent proliferation and survival of the cancer cell [45]. Recent data support a role for SCD1 activity in p53-induced attenuation of AKT signaling *via* modulation of the fatty acid composition of phosphoinositides [46]. Expression of SCD1 is ubiquitous in human tissues, while that of SCD5 is limited. SCD1 was reported to be overexpressed in transcriptome analyses of both HER2-overexpressing breast cancer cells [47] as well as in mucin-1 overexpressing breast cancer cells [48]. Estradiol induces SCD1 expression in ER+ breast cancer cells, MCF7 and T47D [49]. Pharmacologic inhibition of SCD activity reduced cell proliferation in human breast cancer cells MCF-7 and MDA-MB-231 [50]. Expression of SCD1 mRNA is highest in the HER2-enriched subtype in 3 different studies (Table 2).

## UPTAKE OF EXOGENOUS FATTY ACIDS

In addition to *de novo* fatty acid synthesis, cancer cells can acquire needed fatty acids through uptake of dietary lipids or uptake of exogenous fatty acids released by cancer-associated adipocytes (CAA) [51]. A review of pathways exploited by the cancer cell to meet the need for fatty acids can be found in reference [52]. Two of the proteins involved in cellular acquisition of external lipids are lipoprotein lipase (LPL) and the very low density lipoprotein receptor (VLDLR). Intracellular transport of acquired lipids is accomplished by fatty acid binding proteins (FABPs).

### Lipoprotein lipase (LPL)

Although it was originally thought that breast cancer cells did not have access to circulating lipids due to lack of LPL, a more recent study confirmed that basal-like breast cancer cell lines express significant levels of LPL mRNA [53]. In the same study, the majority of breast tumor tissues were positive for LPL when examined immunohistochemically. In our analysis of mRNA expression by tumor tissue shown in Tables 1 and 2, we found preferential expression of LPL mRNA in TNBC and normal-like and basal-like tissue samples.

### Very low density lipoprotein receptor (VLDLR)

VLDLR is a plasma membrane lipoprotein receptor that mediates uptake of exogenous cholesterol and triglycerides. He *et al* found that VLDLR (variant II) protein expression was upregulated in breast tumor *versus* normal tissue and that expression levels were positively associated with lymph node and distant metastases [54]. In our analysis, VLDLR mRNA expression was elevated in TNBC and receptor-negative molecular subtypes (Tables 2 and 3). Silencing of ER in MCF7 cells increased VLDLR mRNA expression (Table 3).

### Fatty acid binding proteins (FABP5 & FABP7)

Fatty acid binding proteins are a family of cytoplasmic proteins that bind long chain fatty acids facilitating uptake into the cell and transport to subcellular organelles. Aberrant expression of FABPs has been associated with a number of disease states, including metabolic disease, non-alcoholic fatty liver disease, cardiovascular disease and cancer [55]. Overexpression of both FABP5 and FABP7 have been associated with TNBC and the basal-like subtype of breast cancer [56–58]. Genetic ablation of FABP5 prevents mammary tumorigenesis in mice [59]. The accumulation of lipid droplets during hypoxia, which is required for survival after reoxygenation, is dependent on expression of FABP7 and FABP3, as well as PLIN2 [60]. Tables 1 and 2 indicate that both FABP5 and 7 mRNAs are overexpressed in TNBC.

## FATTY ACID ACTIVATION

The metabolism of long-chain fatty acids in the synthesis of more complex lipids or as substrates for oxidation processes requires an activation step in the form of thioesterification. While the conversion of arachidonic acid to eicosanoids does not utilize the thioester intermediate directly, recent evidence in breast cancer cells and arterial smooth muscle cells [61, 62] suggests a pathway of arachidonic acid metabolism to prostaglandin E2 (PGE2) that is predicated on the prior storage and subsequent mobilization of arachidonate substrate, thus the conversion of arachidonic acid to arachidonoyl-CoA becomes associated with increased, rather than decreased prostaglandin production.

To date, 5 mammalian ACSL isoforms that differ in subcellular location and substrate specificity have been identified [63]. It has been suggested that ACSL enzymes function as cancer survival factors [64]. ACSL4 is the most extensively studied with respect to the malignant phenotype. ACSL4 has a marked preference for arachidonic acid as substrate and is located primarily in peroxisomes and on the mitochondria-associated membrane as a peripheral, rather than integral, membrane protein [65, 66]. ACSL4 has more recently been localized to activated lipid droplets [67]. With respect to malignant transformation, ACSL4 is overexpressed in hepatocellular carcinoma [68, 69] as well as in colon adenocarcinoma [70], and has been demonstrated to play a role in the more aggressive forms of breast cancer [7, 61, 71–75]. As demonstrated in tables 1 and 2, ACSL4 mRNA is significantly overexpressed in TNBC and basal-like breast cancers, while ACSL3 mRNA expression predominates in RPBC and luminal and HER2-enriched subtypes. Silencing of ER in MCF7 cells results in a 12-fold increase in ACSL4 mRNA, while reducing that for ACSL3 1.21-fold (Table 3).

The differential mRNA expression data for ACSL4 shown in Tables 1-3 have been validated with respect to protein expression [61, 71] as has the role of ACSL4 in mediating the expression of an aggressive phenotype in breast cancer cells [7, 61, 76]. The data further suggest that ACSL4 might function as a potential biomarker for QNBC as well as for hormone resistance when expressed in RPBC. Since forced expression of ACSL4 in breast cancer cells both increases COX-2 protein as well as production of PGE2 [61], it has been suggested that a combinatorial approach of targeting both ACSL4 and COX-2 might comprise an effective therapy for more aggressive breast cancers [72, 76]. A role for the mTOR pathway as well as the lipoxygenase pathway has also been been indicated by recent data [73]. Forced expression of ACSL4 in MCF7 and SKBr3 cells reduces expression of AUTS2 mRNA by 90%, suggesting that this transcription factor may play a role in mammary differentiation [7].

Interestingly, the tyrosine phosphatase, SHP2, that has been demonstrated to increase expression of ACSL4 protein in MA-10 Leydig cells [77], has been shown to promote breast cancer progression [78] and to increase motility of TNBC [79], while inhibition of SHP2 induces a basal-to-luminal transition in breast cancer cells characterized by ER expression, estrogen growth dependency and sensitivity to anti-estrogen therapy [80].

Finally, ACSL4 has been demonstrated to be one of three downstream targets of FOXM1-induced migration in MDA-MB-231 cells [74] as well as a mediator of the tumorigenic role of PADI2 in conferring susceptibility to breast cancer [75].

The very long chain synthetase, SLC27A2 (also known as ACSVL1), a peroxisomal enzyme that activates long-chain fatty acids prior to oxidation, is overexpressed in receptor-positive, luminal subtypes of breast cancer. Silencing of ER in MCF7 cells decreases mRNA expression by 52-fold (Table 3).

## FATTY ACID STORAGE AND MOBILIZATION

Lipid droplets are subcellular organelles that are comprised of a phospholipid monolayer membrane delineating a storage space for fatty acids and cholesterol in the form of triglycerides and cholesterol esters. Data support the hypothesis that lipid droplets play an active role in mediating a variety of inflammatory conditions, including cancer [81]. Nutrient deprivation [82] as well as hypoxia [60], have been shown to induce lipid droplet formation, which in turn increases survival of cancer cells. Included in this organelle are a panoply of proteins with both structural and enzymatic functions. As noted above, both ACSL3 and ACSL4 have been localized to lipid droplets, as has LPIN1 [83]. Lipin 1 (LPIN1) is a phosphatidate phosphatase that catalyzes the conversion of phosphatidic acid to diacylglycerol, the penultimate step in triglyceride synthesis. It also functions as a transcriptional co-activator controlling genes involved in fatty acid oxidation (for review, see [84]). The hypoxia-induced accumulation of lipids in lipid droplets is accompanied by an increase in LPIN1 expression secondary to binding of HIF-1 to the hypoxia response element on the LPIN1 gene promoter, and can be inhibited by silencing the LPIN1 gene [85]. In breast and prostate cancer cell lines, knock down of LPIN1 inhibits proliferation and migration [86]. LPIN1 mRNA is overexpressed in TNBC (Table 1A and 1B), specifically in the basal-like subgroup (Table 2).

Cytosolic PLA2α (PLA2G4A), has been localized to lipid droplets [87–89] along with its substrate, arachidonoyl phosphatidylcholine [87], and has been demonstrated to play a critical role in lipid droplet formation, although the precise mechanism is unclear [82]. The localization of arachidonic acid-metabolizing enzymes, such as COX-2, to lipid droplets suggests that synthesis of prostaglandins, such as PGE2, might take place in a coordinated manner within the lipid droplet domain [90]. Both PLA2G4A as well as COX-2 (not shown) are over expressed in basal-like-TNBC, as well as induced as a result of silencing of ER (Tables 1, 2 and 3).

Mobilization of free fatty acids from triglycerides in lipid droplets is regulated by a class of proteins called perilipins. The perilipins comprise a family of 5 proteins localized to the surface of lipid droplets that regulate access of cellular lipases to stored triglyceride *via* phosphorylation state [91, 92]. As such, they play a regulatory role in the cell's ability to utilize the fatty acids stored in triglycerides. Perilipin 1 (PLIN1) is expressed almost exclusively in adipose tissue, although there is a report of its expression in breast cancer [93]. More recently it has been demonstrated that PLIN1 expression is downregulated in breast cancer, and that reduced expression of PLIN1 in ER+ and luminal A subtypes is associated with a poor prognosis and decreased any event-free overall survival [94]. Exogenous overexpression of PLIN1 in breast cancer cell lines inhibited proliferation, migration, invasion as well as *in vivo* tumorigenesis in mice [94]. PLIN2 expression, on the other hand, has been associated with increased lipid droplet formation and prolonged breast cancer cell survival *in vitro* [95]. PLIN2 mRNA is overexpressed in basal-like-TNBC (Tables 1 and 2), and downregulation of ER increases its expression (Table 3).

For the most part, expression of proteins involved in lipid droplet formation is increased in TNBC and basal-like breast cancers; however, certain proteins present in lipid droplets, such as ACSL3 and PLIN5, are underexpressed in TNBC, suggesting that there are different types of lipid droplets that might function to channel fatty acids to specific metabolic pathways. Along the same lines, mRNA for enzymes that mobilize free fatty acids from triglycerides, including monoglyceride lipase (MGLL) and adipose triglyceride lipase (PNPLA2), appear to be underexpressed in basal-like-TNBC (Tables 1 and 2), suggesting that mobilization of free fatty acids predominates in RNBC. However, expression of MGLL has also been demonstrated to be associated with increased aggressive properties in a variety of cancer cell lines, including breast [96].

## FATTY ACID OXIDATION (FAO)

Although the emphasis with respect to dysregulation of lipid metabolism in cancer has been on lipogenesis and increased expression of lipogenic enzymes such as FASN, it is clear that fatty acid oxidation also plays a role in supporting the malignant phenotype [97]. Fatty acids comprise an efficient source of energy that results in the generation of a large quantity of ATP, and under conditions of nutrient deprivation cancer cell survival depends on FAO [82]. The glucose addiction induced in glioblastoma cells by activation of AKT, for example, can be overcome by activation of FAO [98]. The analysis of the expression of mRNAs associated with fatty acid oxidation as shown in Table 1 suggests that this pathway, like that of *de novo* fatty acid synthesis, is more active in RPBC than in TNBC. This finding is consistent with the hypothesis of Delgoffe and Powell that utilization of fatty acids as fuel in heavily proliferative cells would interfere with membranogenesis, and therefore fatty acid oxidation would preferentially serve as a source of energy in more quiescent cells [99]. Thus in the less highly proliferative RPBC both fatty acid synthesis and oxidation are enhanced, suggesting a futile cycle. It has further been suggested that this futile cycle might function to maintain mitochondrial integrity [100].

The rate-limiting step in the mitochondrial fatty acid β-oxidation pathway is the transport of fatty acyl-CoAs into the mitochondria by carnitine palmitoyl transferase 1 (CPT1) proteins. The meta-analyses shown in Tables 1 and 2 indicate that CPT1A mRNA expression levels are higher in RPBC *versus* TNBC, and predominate in HER2-enriched and luminal B subtypes. There are additional data supporting these observations. Immunohistochemical data indicate the predominance of CPT1A protein in HER2-enriched breast tumors [41]. Using an integrated genomic strategy, Gatza *et al* recently identified CPT1A as a genetic driver of proliferation specifically in the luminal subtype of breast cancer [101]. Another isoform, CPT1C, has been determined to promote cell survival and breast tumor growth under conditions of metabolic stress [102]. In addition to its mitochondrial transport activity, CPT1A has also been demonstrated to localize to the nucleus, where it has been shown to bind to HDAC1 [103]. Silencing of CPT1A expression was correlated with increased apoptosis, inhibition of metastasis, a reduction in HDAC1 activity and hyperacetylation of histones [104].

Predomination of CPT1A expression in RPBC, however, does not preclude a role for FAO in TNBC. In fact, treating MYC-overexpressing TNBC with an inhibitor of CPT1A was effective in decreasing energy metabolism in MYC-overexpressing TNBC cells and in blocking tumor growth in a MYC-driven transgenic TNBC model as well as in a MYC-overexpressing patient-derived xenograft [16]. And under conditions of chronic acidosis, glucose utilization is decreased as a source of acetyl-CoA, while uptake of exogenous fatty acids and FAO is increased [105].

Fatty acid oxidation can also take place in peroxisomes. Branched chain acyl-CoA oxidase 2 (ACOX2) is the rate-limiting enzyme in the β-oxidation of branched, long-chain fatty acids that takes place in peroxisomes. Expression and translation of a variant transcript, ACOX2-i9, has been demonstrated to be regulated by estrogens in ER-positive breast cancer cells lines, and knock down of this enzyme results in decreased cell viability [106]. Although ACOX2 mRNA is overexpressed in RPBC and luminal subtypes, paradoxically, silencing of ER results in increased expression of ACOX2 mRNA (Table 3).

## CONCLUSIONS

Data summarized here indicate that dysregulation of fatty acid metabolism is essential for maintenance of proliferation and survival of breast cancer cells. To date, potential treatment options have overwhelmingly targeted FASN, and the first-in-human investigation of the oral first-in-class FASN inhibitor, TVB-2640, is currently underway (https://clinicaltrials.gov/show/NCT02223247); however, several other lipid metabolic enzymes have known inhibitors, some even FDA approved (for a list, see [107]). For example, targeting ACACA has the advantage that, since this enzyme has long been considered a target in the treatment of metabolic diseases, there are a number of inhibitors already available (for review see [108]). Likewise, inhibitors are also available for the rate-limiting enzyme in FAO, CPT1A [109]. One of these inhibitors, ranolazine, has FDA approval. Inhibiting fatty acid activation, on the other hand, has the advantage of blocking both synthesis and oxidation, as well as overcoming the need to inhibit uptake of exogenous fatty acids. Triacsin C is a fatty acid analogue that has been shown to inhibit ACSL 1, ACSL3 and ACSL4. This compound causes apoptosis of many cancer cell types, including breast [71, 110–113], and has been demonstrated to inhibit the growth of xenograft tumors [110]. Lipid droplets comprise an emerging target in a number of diseases, including cancer, and it has been suggested that the lipid droplet protein, PLIN2, might make an effective target [114]. Based on the differential expression of FAM proteins with respect to intrinsic molecular subtype in breast cancer, the design of future studies needs to account for this heterogeneity.
